# Recent developments in maximum likelihood estimation of MTMM models for categorical data

**DOI:** 10.3389/fpsyg.2014.00269

**Published:** 2014-04-08

**Authors:** Minjeong Jeon, Frank Rijmen

**Affiliations:** ^1^Department of Psychology, Ohio State UniversityColumbus, OH, USA; ^2^CTB/McGraw-Hill, Psychometric ServicesMonterey, CA, USA

**Keywords:** multitrait-multimethod model, crossed factors, maximum likelihood estimation, variational maximization-maximization, alternating imputation posterior, monte carlo local likelihood

## Abstract

Maximum likelihood (ML) estimation of categorical multitrait-multimethod (MTMM) data is challenging because the likelihood involves high-dimensional integrals over the crossed method and trait factors, with no known closed-form solution. The purpose of the study is to introduce three newly developed ML methods that are eligible for estimating MTMM models with categorical responses: Variational maximization-maximization (e.g., Rijmen and Jeon, 2013), alternating imputation posterior (e.g., Cho and Rabe-Hesketh, 2011), and Monte Carlo local likelihood (e.g., Jeon et al., under revision). Each method is briefly described and its applicability for MTMM models with categorical data are discussed.

## 1. Introduction

The multitrait-multimethod (MTMM) design is an important methodological tool for investigating the construct validity (convergent and discrimination validity) of psychological measures. The advantages of applying confirmatory factor analysis (CFA), or more broadly, structural equation models (SEM) has been widely recognized for the analysis of MTMM data (e.g,. Widaman, 1985; Marsh and Hocevar, 1988; Marsh, 1989; Marsh and Grayson, 1995; Dumenci, 2000; Eid et al., 2006). For instance, SEM allows measurement error to be separated from method-specific effects and tests the nature of trait and method influences (Nussbeck et al., 2006).

Traditional applications of SEM to MTMM data are based on continuous outcome variables which was required by traditional SEM software (e.g., Joreskog and Sorbom, 1984). However, psychological inventories often employ categorical response categories based on a Likert scale. Researchers usually aggregate the item-level categorical responses to create (sub)test-level continuous outcomes. However, this leads to an undesirable loss of information at the item level that could be useful for test construction. For example, researchers could be interested in choosing only those items with high convergent and discriminant validity coefficients in establishing a test (Nussbeck et al., 2006).

Typical SEM MTMM models include multiple traits and methods that are treated as latent variables (or factors, random effects). Multiple traits are needed in order to estimate the discriminant validity of represented constructs and the degree to which observed scores measure the traits under consideration; multiple methods are needed to evaluate the impact of different methods on the observed scores, that is, to which degree observed scores are influenced by the way they are measured (i.e., rater biases or biases due to the use of different scales for the same constructs). Trait and method factors are cross-classified (or crossed) with each other in the sense that a set of different traits are measured by the same set of methods. The cross structure creates major challenges in maximum likelihood (ML) estimation. In particular, with categorical responses, the ML computation involves numerical integration over high dimensional intractable integrals over the crossed latent variables. For example, when integrals over method and trait factors are evaluated using Gaussian quadrature (e.g., Bock and Aitkin, 1981), the number of evaluation points increases exponentially with the total number of latent variables (i.e., method + trait factors). Even though the number of quadrature points can be reduced with adaptive quadrature (e.g., Pinheiro and Bates, 1995; Rabe-Hesketh et al., 2005), the total number of evaluation points still increases exponentially with the number of latent variables. In addition, adaptive quadrature involves the computation of the posterior mode and curvature at the mode of the latent distribution for each response pattern, whose complexity also increases with the number of latent variables (Rijmen, 2009).

Limited information techniques have been adopted to estimate SEM models for categorical data (e.g., Browne, 1984; Bollen, 1989; Satorra, 1989, 1992; Joreskog, 1994; Muthen et al., unpublished manuscript). Unlike full ML estimation methods, limited information ML methods do not take into account the complete joint contingency table of all items, but only marginal tables up to the fourth order (Mislevy, 1986). Weighted least squares estimation is then carried out, which is reasonably fast even for high-dimensional models. However, the number of elements in the optimal weight matrix, which has to be invertible, grows with the fourth power of the number of indicators (Mislevy, 1986); accordingly, SEM MTMM models with multiple indicators may require a huge sample size, which may be impractical in most psychological applications (Rijmen, 2009).

Alternatively, Muthen et al. (unpublished manuscript) proposed a robust weighted least squares method (WLSMV) where the optimal weight matrix is replaced by a diagonal matrix. The performance of WLSMV has been evaluated in the context of simple structure CFA models (Yu, 2002; Beauducel and Herzberg, 2006) and of a longitudinal population model (Muthen et al., unpublished manuscript). Recently, Nussbeck et al. (2006) performed a simulation study to evaluate the performance of WLSMV on CT-C(M-1) models for ordinal responses and showed that WLSMV works quite well with adequate sample sizes. Still, the quality of WLSMV and its data requirements have not been thoroughly established in a variety of situations and for more complex SEM MTMM models.

In principle, estimation methods that are developed for item response theory (IRT) models can be applied to MTMM models for categorical data since many SEM models can be parameterized as IRT models (e.g., Muthen, 1978; Takane and de Leeuw, 1987). For ML estimation of complex IRT models, Monte Carlo (MC) methods have been widely utilized to approximate the likelihood (or the posterior), e.g., single sample methods such as stochastic EM (e.g., Ip, 1994) or metropolis-Hastings Robbins-Monro (MH-RM) (e.g., Cai, 2010), and multiple sample methods such as MCEM (e.g., McCulloch, 1997). Single sample methods are fast but highly depend on initial values of model parameters whereas multiple sample methods can be computationally slow for complex problems. In addition, a modified expectation-maximization (EM) algorithm has been developed which uses a sequence of integrations over subsets of latent variables in the E-step to estimate highly complex IRT models (Boeck, 2008; Rijmen, 2009; Jeon et al., 2013b). However, its computational complexity remains high for MTMM types of models because the latent-space cannot be decomposed into low-dimensional sub-spaces due to the crossed structure of the latent variables (for details, see Rijmen and Jeon, 2013).

This paper introduces three recent developments in ML estimation of IRT models with crossed random effects structures: (1) Variational maximization-maximization (MM; e.g., Rijmen and Jeon, 2013), (2) alternating imputation posterior (AIP; e.g., Cho and Rabe-Hesketh, 2011), and (3) Monte Carlo local likelihood (MCLL; e.g., Jeon et al., under revision). We provide a brief review of each method and discuss the applicability of each method for estimating MTMM models for categorical indicators.

The rest of this paper is organized as follows: Section 2 describes a SEM MTMM model that is considered in this paper. Section 3 provides a description of the three estimation methods. In Section 4, an empirical illustration will be provided using the MCLL method as an example. This paper ends with some concluding remarks in Section 5.

## 2. MTMM model

Typical SEM MTMM models contain multiple traits (e.g., depression and anxiety) measured by multiple methods (e.g., self, teacher, and peer ratings). In statistical terms, traits and methods are two latent variables (or factors) that are crossed with each other. Therefore, the models can be applied to cases where multiple latent variables of two kinds are present in a cross-classified factorial design.

As an illustration, we consider a SEM MTMM model with correlated trait factors and uncorrelated method factors (CT-UM). We focus on binary variables, but an extension to polytomous variables is straightforward.

Suppose total *I* binary indicators are observed for person *p* (*p* = 1,…,*N*) for *T* continuous trait factors *θ*^*T*^_*pt*_ (*t* = 1,…,*T*) and *M* method factors *θ*^*M*^_*pm*_ (*m* = 1,…,*M*). The conditional probability of a correct response (or response being 1) to indicator *i*, π_*pi*_ = *p*(*y_pi_* = 1|*θ*^*T*^_*pt*_, *θ*^*M*^_*pm*_), can then be written as

(1) $$g\left(\pi_{pi}\right)=\alpha_{it}^{T}\theta_{pt(i)}^{T}+\alpha_{im}^{M}\theta_{pm(i)}^{M}+\beta_{i},$$

where *g*(·) is the link function, α^*T*^_*it*_ is the loading for indicator *i* for the *t*th trait factor *θ*^*T*^_*pt(i)*_ that indicator *i* belongs to, α^*M*^_*im*_ is the loading for indicator *i* for the *m*th trait factor *θ*^*T*^_*pm(i)*_ that indicator *i* belongs to, and β_*i*_ is the intercept (or location) for indicator *i*. For link function *g*(·), a logit or probit link is typically used for binary responses. For polytomous responses, the cumulative logit link or the adjacent-category logit link can be used. Variances of all latent variables are fixed to 1 for factor standardization (and all factor loadings are estimated). In each combination of method and trait factors, more than one indicator variables can be allowed. The CT-UM model in (1) assumes that trait factors are correlated with each other whereas method factors are uncorrelated with each other and with trait factors; that is, *θ*^*T*^_*pt*_ ~ *N*(0, Σ^*T*^) and *θ*^*M*^_*pm*_ ~ *N*(0, *I*), where *I* is an identity matrix and the diagonal elements of Σ^*T*^ are 1.

Model (1) assumes that the variance of observed data is additively decomposed into multiple variance components involved with trait factors *θ*^*T*^_*pt*_ and method factors *θ*^*M*^_*pm*_. This allows us to define the consistency and method-specificity coefficients as the proportion of the true variance (without error) to variance determined by trait and method factors, respectively:

(2) $$\gamma_{pi}^{T}=\frac{\alpha_{it}^{T}\text{Var}\left(\theta_{pt}^{T}\right)}{\alpha_{it}^{T}\text{Var}\left(\theta_{pt}^{T}\right)+\alpha_{im}^{M}\text{Var}\left(\theta_{pm}^{M}\right)},$$

(3) $$\gamma_{pi}^{M}=\frac{\alpha_{im}^{M}\text{Var}\left(\theta_{pm}^{M}\right)}{\alpha_{it}^{T}\text{Var}\left(\theta_{pt}^{T}\right)+\alpha_{im}^{M}\text{Var}\left(\theta_{pm}^{M}\right)},$$

where γ^*T*^_*pi*_ is the consistency coefficient and γ^*M*^_*pi*_ is the method-specificity coefficient. The consistency coefficient γ^*T*^_*pi*_ can also be seen as evidence of convergent validity (Nussbeck et al., 2006).

Note that model (1) is equivalent to an IRT model with two crossed latent variables. Rost and Carstensen (2002) presented such a model with two crossed latent traits that represent item contents and contexts, respectively. In their multidimensional facet model, the factor loadings (or discrimination parameters) were fixed to 1, and a joint maximum likelihood (JML) method was used for estimation; however, JML is known to produce inconsistent parameter estimates for a finite number of items regardless of the person sample size (Neyman and Scott, 1948; Andersen, 1970; Ghosh, 1995). Jeon et al. (2013a) presented a bifactor extension of the MTMM IRT model where a general factor is incorporated in addition to the method and trait factors. The method and trait factors are assumed to be independent of each other conditional on the general factor. In addition, with fixed factor loadings, model (1) can be seen as a generalized linear mixed model with crossed random effects. Such a model has been widely utilized in psychometrics e.g., for investigating random item effects (e.g., De Boeck, 2008; Cho et al., 2014).

The computational complexity of estimating model (1) can be shown by writing down its likelihood function

(4) $$\begin{array}{l} L\left(y;\Psi \right)=\int_{\theta_{1}^{T}}\cdots \int_{\theta_{T}^{T}}\int_{\theta_{1}^{M}}\cdots \int_{\theta_{M}^{M}}p\left(y|\theta^{T},\theta^{M}\right)\left(\prod_{t}p\left(\theta_{t}^{T}\right)\right)\\ \;\left(\prod_{m}p\left(\theta_{m}^{M}\right)\right)d\theta_{M}^{M}\cdots d\theta_{1}^{M}d\theta_{T}^{T}\cdots d\theta_{1}^{T}, \end{array}$$

where **y** is the vector of responses, **Ψ** the vector of all parameters, **Ψ** = (**α**^*T*^, **α**^*M*^, **β**,)′, *p*(*θ*^*T*^_*t*_) and *p*(*θ*^*M*^_*m*_) are the prior distributions for *θ*^*T*^_*t*_ and *θ*^*M*^_*m*_, and *p*(**y**|*θ*^*T*^, *θ*^*M*^) is the joint probability of all observed responses given the latent variables where *θ*^*T*^ = (*θ*^*T*^_1_,…, *θ*^*T*^_*T*_)′ and *θ*^*M*^ = (*θ*^*M*^_1_,…, *θ*^*M*^_*M*_)′, and

$$p\left(y|\theta^{T},\theta^{M}\right)=\prod_{m}\prod_{t}p\left(y_{ip}|\theta_{t}^{T},\theta_{m}^{M}\right).$$

The multiple integrals in Equation (2) have no closed form solution and require numerical integration, which is challenging with regular quadrature methods. For example, to estimate a model with three method and three trait factors, even with a moderate amount of eight quadrature points, a total of 262, 144 (= 8^6^) evaluations are required with Gaussian quadrature, which is prohibitive in practical settings.

## 3. Estimation methods

In this section, we describe three recent developments in ML estimation of latent variable models with crossed factors for categorical data.

### 3.1. Variational maximization-maximization (MM)

The variational maximization-maximization (MM) algorithm (Rijmen and Jeon, 2013; Jeon et al., under revision) is a modified version of the EM algorithm (Dempster et al., 1977). In the traditional E-step, the expectation of the complete data log-likelihood, log *f*(**y**, ***θ***; **Ψ**) is computed over the posterior distribution of the latent variables ***θ*** (or missing data) given the observed data **y** and given current parameter estimates. For instance, the expectation (or *Q* function) can be defined at the *m*th iteration as

$$\begin{array}{l} Q\left(\Psi ;\Psi^{\left(m\right)}\right)=E\left\{\text{log}f\left(y,\theta ;\Psi \right)|y;\Psi^{\left(m\right)}\right\}\\ \;=\int_{\theta }p\left(\theta |y;\Psi^{\left(m\right)}\right)\text{log}f\left(y,\theta ;\Psi \right)d\theta , \end{array}$$

where **Ψ**^(*m*)^ are the current parameter estimates and *p* (***θ***|**y**; **Ψ**^(*m*)^) is the probability density of the latent variables given the data for the current parameter estimates. The challenge is that the *Q* function cannot be evaluated analytically due to the integral over the posterior distribution *p*(***θ***|**y**; **Ψ**^(*m*)^).

The variational MM algorithm replaces the posterior distribution *p*(***θ***|**y**; **Ψ**^(*m*)^) by a tractable alternative probability density function *g*(***θ***), which is called a variational density. The variational density function *g*(***θ***) is found by minimizing the Kullback-Leibler (KL) divergence (Shorack and Wellner, 1986, p.159) from *g*(***θ***) to *p*(***θ***|**y**; **Ψ**^(*m*)^). It can be shown that minimizing the *KL* is equivalent to maximizing a lower bound of the log-likelihood (Bishop, 2006).

The MM algorithm involves two maximizations: The first M-step that maximizes the lower bound *l* (**y**; **Ψ**^(*m*)^) with respect to *g*(***θ***) given the current parameter estimates **Ψ**^(*m*)^ and the second M-step that maximizes *l* (**y**; **Ψ**) with respect to **Ψ** given the current variational approximation *g*(***θ***).

In the variational MM-algorithm, the variational density function *g*(***θ***) should be chosen close to the true model-based posterior distribution *p* (***θ***|**y**; **Ψ**) and make the integrals computationally tractable. The mean-field approximation has been adopted to approximate *g*(***θ***) (Rijmen and Jeon, 2013; Jeon et al., under revision), which assumes complete factorizability (or independence) of the latent variables *θ* under the posterior (Hall and Tao, 2002; Bishop, 2006); that is, *g*(***θ***) = ∏_*i*_*g*_*i*_ (*θ*_*i*_), where *θ*_*i*_ is the *i*th element of *θ* and *g*_*i*_ (*θ*_*i*_) is the corresponding marginal density.

The variational technique was introduced to psychometrics by Humphreys and Titterington (2003), but first applied by Rijmen and Jeon (2013) to estimate a complex IRT model for random item parameters across countries using discrete random effects. The variational MM algorithm was later extended by Jeon et al. (under revision) for continuous random effects and included adaptive quadrature. Jeon et al. (under revision) and Rijmen et al. (in press) successfully applied the algorithm to estimate IRT models with random item difficulty parameters.

It has been shown that the variational MM algorithm generally performs as well as the Laplace approximation (Tierney and Kadane, 1986; Lindstrom and Bates, 1988; Wolfinger, 1993) which works well in most situations (Joe, 2008). With small cluster sizes and large variance components, where the Laplace approximation is known to perform poorly, the variational MM algorithm performed better than the Laplace approximation (Jeon et al., under revision). The variational algorithm can be applied to estimate MTMM models with correlated traits and/or correlated method factors when the factor loadings are fixed to known values. However, this algorithm has not yet been applied to estimate models with loading parameters. In addition, the variational approximation based on the full factorization of latent variables may not be applicable for MTMM models with correlated trait-method factors. Therefore, further research is required for applying the variational MM algorithm to estimate various MTMM models.

### 3.2. Alternating imputation posterior (AIP)

The key goal of the alternating imputation posterior (AIP) algorithm is to lower computational costs by splitting the ‘random part’ of the model (that involves latent variables) into several pieces that involve a small number of latent variables, which correspond to wings in the algorithm. For instance, the random part (α^*T*^_*it*_ *θ*^*T*^_*pt(i)*_ + α^*M*^_*im*_*θ*^*M*^_*pm(i)*_) in model (1) can be divided into two wings, the trait wing that includes α^*T*^_*it*_ *θ*^*T*^_*pt(i)*_ and the method wing that includes α^*M*^_*im*_*θ*^*M*^_*pm(i)*_. The algorithm alternates the multiple wings where estimation is carried out by holding the other latent variables constant (Cho and Rabe-Hesketh, 2011). Specifically, computation within a wing consists of two steps: imputation (I) and posterior (P) steps. In the I-step, latent variables (or missing data) are imputed by sampling from the posterior distribution given the observed data. The P-step updates the approximation of the posterior distribution.

Clayton and Rasbash (1999) first presented the AIP algorithm using marginal quasi-likelihood (MQL; Goldstein, 1991) and penalized quasi-likelihood (PQL; Breslow and Clayton, 1993) for computation within a wing. However, MQL and PQL are known to underestimate variance parameters (Cho and Rabe-Hesketh, 2011). Cho and Rabe-Hesketh (2011) proposed an improved AIP algorithm by replacing MQL/PQL with adaptive quadrature (Pinheiro and Bates, 1995; Rabe-Hesketh et al., 2005).

For simplicity, here the AIP algorithm is illustrated using a simpler version of model (1) that assumes independent trait and method factors. First, we define the trait and method wings that include the trait and method factors, respectively. By assuming the other factor and its factor loadings as known, each wing estimates a two parameter logistic (2PL) IRT model. For example, in the trait factor wing at the *k*th iteration, given α^*M*(*k* − 1)^_*im*_ and *θ^M(k − 1)^_pm(i)_* fixed to the values from (*k* − 1) iteration, the following 2PL model is estimated

$$g\left(\pi_{pi}\right)=\alpha_{it}^{T}\theta_{pt(i)}^{T}+\alpha_{im}^{M(k-1)}\theta_{pm(i)}^{M(k-1)}+\beta_{i},$$

where the item parameters are Ψ^(*k*)^ = (α^*M(k)*^_*im*_, β^(*k*)^_*i*_), *i* = 1,… I and their covariance is $\Sigma_{\Psi }^{\left(k\right)}$. Then, the item parameters $\hat{\Psi }$ are sampled from the normal distribution

$$\Psi^{\left(k\right)}|\theta_{pm(i)}^{M^{\left(k-1\right)}},\theta_{pt(i)}^{T^{\left(k-1\right)}}\sim N\left({\hat{\Psi }}^{\left(k\right)},{\hat{\Sigma }}_{\Psi }^{\left(k\right)}\right).$$

Finally, person random effects *θ*^*T*^_*1t(i)*_, …, *θ*^*T*^_*Nt(i)*_ are sampled from a normal approximation to its conditional posterior distribution (using posterior means and variances). Given the estimates α^*T*(*k* − 1)^_*it*_, *θ*^*T*(*k* − 1)^_*pt(i)*_, the algorithm moves to the method wing that estimates α^*M(k)*^_*im*_ and β^(*k*)^_*i*_. This sequence alternates until convergence.

An important advantage of the AIP algorithm is that it can be easily adapted to estimate other complex random effects models with minimal programming. Any software can be used which provides an option for specifying a variable to be added to the linear predictor without estimating a corresponding regression coefficient. For instance, Cho and Rabe-Hesketh (2011) and Cho et al., (2014) implemented the AIP algorithm using xtmelogit and gllamm in Stata (StataCorp, 2009) and applied it to estimate IRT models with random item difficulty and with random item difficulty and discrimination parameters.

However, the AIP algorithm may not be beneficial for models whose random parts are not readily decomposed into smaller pieces. For example, the MTMM model with correlated trait factors requires the trait wing to estimate a multidimensional 2PL IRT model, which may be computationally demanding with a large number of trait factors. With correlated method-trait factors, it is impossible to split the latent variables into smaller parts; therefore, the AIP algorithm provides no additional benefits.

### 3.3. Monte Carlo local likelihood (MCLL)

Monte Carlo local likelihood (MCLL) (Jeon et al., under revision) is an approximate ML method using Monte Carlo samples of model parameters. MCLL approximates the likelihood function as the local likelihood estimate of the posterior density divided by the prior density where the local likelihood estimate of the posterior density is obtained by approximating the log-posterior density with a polynomial function. Specifically, MCLL begins with generating Markov chain Monte Carlo (MCMC) samples of model parameters from the posterior for a particular prior distribution

$$p\left(\theta |y\right)=\frac{L\left(y|\theta \right)p\left(\theta \right)}{C_{s}},$$

where *p*(***θ***|**y**) is the posterior, *L*(**y**|***θ***) is the likelihood, *p* (***θ***) is the prior, and *C*_*s*_ is the normalizing constant, *C*_*s*_ = ∫ *L*(**y**|***θ***)*p*(***θ***)*d**θ***.

The likelihood function is approximated up to a constant by fitting a density to the MCMC samples and dividing it by the prior

(5) $$\hat{L}\left(y|\theta \right)=\frac{1}{p\left(\theta \right)}{\text{Ps}}_{p}\left(\theta |y\right),$$

where *Ps*_*p*_ (***θ***|**y**) is the local likelihood estimate of the posterior density, which is obtained for a given value of *θ*, by assuming that the log-posterior density can be locally approximated by a polynomial function. Specifically, a localized log-likelihood for *p*(***θ***|**y**) is defined as

(6) $$\hat{l}\left(\theta \right)=\sum_{j=1}^{m}K_{h}\left(\theta^{\left(j\right)}-\theta \right)\text{log}p\left(\theta^{\left(j\right)}|y\right)-m\int K_{h}\left(u-\theta \right)p\left(u|y\right)du,$$

where *K*_**h**_(·) represents a symmetric unimodal density (or kernel function) whose argument is divided by the corresponding element of **h**, a vector of bandwidths. Here a local polynomial approximation is obtained by assuming *log* *p*(*θ*^(*j*)^|**y**) can be well approximated by a low-degree polynomial in a neighborhood of the fitting point *θ* as

$$\text{log}p\left(\theta^{\left(j\right)}|y\right)\approx P_{a}\left(\theta^{\left(j\right)}-\theta \right),$$

where ***a*** are the parameters of the local polynomial function and estimated for a particular ***θ*** by maximizing a localized version of the log-likelihood in Equation (6).

In principle, the MCLL algorithm can estimate any complex random effects model that is feasible with MCMC but not possible with ML. However, computational costs of the algorithm increase with the total number of model parameters, rather than with the number of latent variables (which is the case for most ML methods). That is, the MCLL estimation can be hindered by a large number of fixed effects model parameters rather than complex random effects structures. For example, the MTMM models with correlated trait-method factors may be feasible with the MCLL algorithm, whereas simple unidimensional IRT models but with a large number of items may not be.

## 4. Illustration

In this section, we apply the MCLL algorithm to estimate the CT-UM model presented in (1). As explained in Section 3.1 and 3.2, the variational MM algorithm and the AIP algorithm are not applicable to estimate model (1) due to the presence of free factor loading parameters and correlations between trait factors, respectively.

The empirical illustration is based on the verbal aggression dataset from De Boeck and Wilson (2004); Vansteelandt (2000). The data come from 316 first-year psychology students (243 females and 73 males), presented with a verbal aggression inventory with 24 items. The inventory concerns the source of verbal aggression (type of situation), the kind of verbally aggressive behavior, and its possible inhibition. Specifically, each item consists of one of four frustrating situations (bus, train, store, and operator), two of which are other-to-blame and two of which are self-to- blame, followed by one of three verbally aggressive behaviors (cursing, scolding, and shouting), and phrased in one of two behavioral modes (wanting and doing). An example item is “A bus fails to stop for me. I would want to curse.”, which corresponds to the “other-to-blame” situation related to “bus,” “cursing” aggressive behavior, and “wanting” behavior mode. “A bus fails to stop for me. I would actually curse” corresponds to the same “other-to-blame” situation related to “bus” and “cursing” aggressive behavior, but “doing” behavior mode. The items include three response categories: No, Perhaps, and Yes. The responses were dichotomized by combining Perhaps with Yes categories.

**Figure 1:**
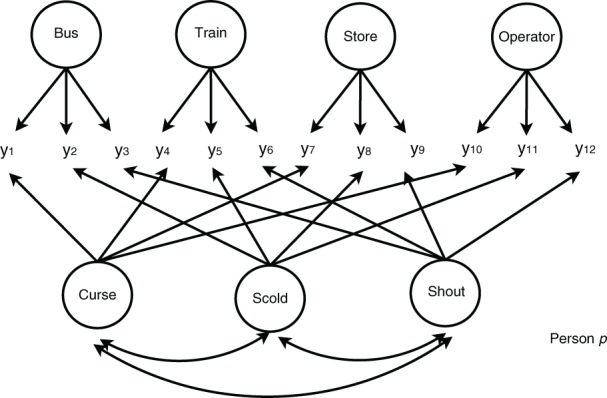
**A multitrait-multimethod model**. *y*_1_ to *y*_12_ are the binary responses for person *p*. Curse, Scold, and Shout are the three factors in the behavior type. Bus, Train, Store, and Operator are the four factors in the situation type.

For simplicity, we used the 12 items that correspond to the “wanting” behavior mode, under four frustrating situation types (bus, train, store, and operator) and three aggressive behavior types (cursing, scolding, and shouting) The situation types and behavior types can be treated as two types of latent variables (or factors). In addition, these two types of factors are crossed with each other because the items under the same frustrating situation types are used to measure different aggressive behavior types. Therefore, the CT-UM model discussed in Section 2 can be applied to analyze this dataset by treating one of the factors (e.g., aggressive behavior types) as trait factors and the other (e.g., frustrating situation types) as method factors. Note that the choice of trait and method factors is arbitrary in this example. We then assume that the trait factors are correlated with each other whereas the method factors are uncorrelated with each other and with trait factors. Figure 1 illustrates the model for person *p*.

In the figure, the frame represents person *p*, circles represent latent variables, and arrows represent connecting latent and/or observed variables represent regression relations. There are three factors for aggressive behavior types (as trait factors) and four factors for situation types (as method factors). The trait factors and method factors are crossed with each other as shown.

The MCLL algorithm was applied to estimate the CT-UM model as follows: First MCMC samples of the model parameters were obtained using the freely available Bayesian software, WinBUGS 1.4 (Lunn et al., 2000). Slightly informative priors were used based on three chains that were obtained from 4000 iterations after 3000 burn-in. An R package mcll (Jeon et al., 2013a) was then used to obtain the MCLL estimates.

Table 1 lists the parameter estimates of the MTMM model. The estimated item intercepts represent the easiness of the items (or minus the difficulties). The results showed that the item difficulties depend both on the situation type and behavior type. Specifically, the Curse items tended to be more difficult than the other behavior type items. The Scold items were more difficult than the Shout items.

**Table 1:** **Parameter estimates of the MTMM model for the verbal aggression data**.

	**Situation**				**Behavior**		
**Item**	**Intercept**	**Bus**	**Train**	**Store**	**Operator**	**Curse**	**Scold**	**Shout**
i1	−1.89	1.62				1.31		
i2	−1.02	1.97					1.45	
i3	−0.18	1.55						1.43
i4	−2.52		1.33			1.13		
i5	−1.36		1.51				1.70	
i6	−0.06		1.56					1.93
i7	−0.67			1.59		0.91		
i8	1.06			2.13			0.70	
i9	2.03			1.56				0.83
i10	−1.40				1.54	0.84		
i11	0.57				1.99		0.85	
i12	1.37				1.31			0.96
Cor_12_						0.21		
Cor_13_						0.12		
Cor_23_						0.86		

The estimated factor loadings tended to be larger for the situation type than for the behavior type for all items except for Scold (item 5) and Shout (item 6) in the Train situation. Specifically, for the Bus situation, the Scold items showed a larger loading than the Curse and Shout items, and for the Train situation, Shout and Scold items showed larger loadings than the Curse item. For the Store and Operator situations, the Scold item showed a larger loading than the Curse and Shout items. For the Curse items, the Bus item showed the largest loading, followed by the Train, Store, and Operator situation items in order. For the Scold and Shout items, the Train and Bus items showed larger loadings than the Operator and Store items. The correlations between the behavior types were estimated as 0.21 between Curse and Scold (Cor_12_), 0.12 between Curse and Shout (Cor_13_), and 0.86 between Scold and Shout modes (Cor_23_).

We computed the proportion of the variance for each item, determined by the behavior type and the situation type as described in Equations (2) and (3), which correspond to the consistency coefficients (γ^*T*^_*pi*_) and the method-specificity (γ^*M*^_*pi*_) coefficients, respectively. The result is presented in Table 2.

**Table 2:** **Coefficients γ^*T*^_*pi*_ and γ^*M*^_*pi*_, where *T* represents the behavior type and *M* represents the situation type**.

	**Situation**				**Behavior**		
**Item**	**Bus**	**Train**	**Store**	**Operator**	**Curse**	**Scold**	**Shout**
i1	0.898				0.102		
i2	0.449					0.551	
i3	0.318						0.682
i4		0.894			0.106		
i5		0.348				0.652	
i6		0.258					0.742
i7			0.926		0.074		
i8			0.646			0.354	
i9			0.447				0.553
i10				0.929	0.071		
i11				0.584		0.416	
i12				0.370			0.630

Table 2 shows that the responses to Curse items tended to be largely determined by the situation type rather than the behavior type. Shout items tended to be more influenced by the behavior type than by the situation type. For Scold items, both the situation type and behavior type appeared to make similar impacts. For the Bus and Train scenarios, the behavior type had slightly larger effects and for the Store and Operator scenarios, the situation type had somewhat larger effects.

## 5. Concluding remarks

Applications of MTMM models for categorical indicators have been limited due to the estimation difficulties and thus a lack of available software. The challenges arise from the crossed structure of the latent variables or random effects (i.e., method and trait factors) whose ML estimation requires high-dimensional numerical integration to evaluate the likelihood function.

This study introduced three novel ML methods, variational ML, AIP, and MCLL algorithms that have recently been developed to estimate crossed random effects models. The key idea of the variational algorithm is to lower the computational burden by factorizing the complex joint posterior distribution of latent variables into a product of low dimensional distributions. Similarly, the AIP algorithm lowers the computational costs by decomposing the latent variables into several smaller pieces so that the actual computation involves only lower-dimensional problems. These methods can be a promising solution for some complex SEM models, but for other models whose decomposition of latent variables is infeasible, such as MTMM models with correlated method and trait factors, the benefits using these algorithms may not be substantial.

The MCLL algorithm has an advantage compared to these two methods given that its computational efficiency does not rely on the factorization of latent variables. Instead, the computational costs increase with the total number of model parameters. Therefore, the algorithm may be applied to estimate complex MTMM models with correlated method and traits but with few items.

This review suggests that these methods could be useful alternatives to the limited information techniques under some circumstances. Therefore, further studies are needed to evaluate the performance of these methods under various modeling specifications and data conditions. This will provide important information for applied researchers in choosing proper computational tools for estimating MTMM models with categorical data.

### Conflict of interest statement

The authors declare that the research was conducted in the absence of any commercial or financial relationships that could be construed as a potential conflict of interest.
